# Diagnostic Utility of a Mycobacterium Multiplex PCR Detection Panel for Tuberculosis and Nontuberculous Mycobacterial Infections

**DOI:** 10.1128/spectrum.05162-22

**Published:** 2023-04-24

**Authors:** Yoshifumi Uwamino, Akio Aono, Yu Tomita, Kozo Morimoto, Masahiro Kawashima, Hirofumi Kamata, Yuka Sasaki, Hideaki Nagai, Naoki Hasegawa, Satoshi Mitarai

**Affiliations:** a Department of Laboratory Medicine, Keio University School of Medicine, Tokyo, Japan; b Department of Infectious Diseases, Keio University School of Medicine, Tokyo, Japan; c Department of Mycobacterium Reference and Research, The Research Institute of Tuberculosis, Japan Anti-Tuberculosis Association, Tokyo, Japan; d Medical & Biological Laboratories Co., Ltd. (MBL), Tokyo, Japan; e Department of Pulmonary Medicine, Fukujuji Hospital, Japan Anti-Tuberculosis Association, Tokyo, Japan; f Department of Pulmonary Medicine, National Hospital Organization Tokyo National Hospital, Tokyo, Japan; g Division of Pulmonary Medicine, Department of Internal Medicine, Keio University School of Medicine, Tokyo, Japan; University of Chicago

**Keywords:** *Mycobacterium tuberculosis*, PCR-rSSO, multiplex PCR, nontuberculous mycobacteria

## Abstract

The prevalence of nontuberculous mycobacterial diseases is increasing worldwide, and tuberculosis remains highly prevalent. Rapid and accurate microbial diagnoses of both tuberculosis and nontuberculous mycobacterial infections are required. A novel PCR–reverse sequence-specific oligonucleotide probe (PCR-rSSO) method-based mycobacterial detection panel (Myco-Panel) test was developed for the rapid identification of 30 mycobacterial species and subspecies. Clinical respiratory samples were collected from patients with suspected or confirmed tuberculosis and nontuberculous mycobacterial lung disease and those with other respiratory diseases. Myco-Panel tests were performed on the samples, and liquid mycobacterial culture and identification were performed for reference according to housekeeping gene sequences of mycobacteria in positive culture tubes. Furthermore, to assess the detection performance for several mycobacterial species rarely recovered in Japan, the accuracy of the Myco-Panel test was investigated using stock mycobacterial type strains and clinical isolates. A total of 178 clinical respiratory samples were analyzed. The Myco-Panel and sequence-based identification results for mycobacterial cultures were 83.1% concordant (kappa coefficient, 0.785 [95% confidence interval, 0.716 to 0.854]). The Myco-Panel correctly identified 281 of the 283 type strains and clinical isolates tested. The Myco-Panel test could accurately detect several mycobacterial species from clinical respiratory samples and mycobacterial suspensions. Rapid and accurate identification of pathogens using respiratory samples is possible using the Myco-Panel.

**IMPORTANCE** Species identification is important for the diagnosis of mycobacterial infections and decisions on treatment regimens. The Myco-Panel test accurately detects clinically common mycobacterial species that cause respiratory infections from clinical respiratory samples and mycobacterial suspensions. The rapid identification of multiple mycobacterial species will provide clinically useful information for the management of patients. Although we understand that the current diagnostic criteria require mycobacterial culture results in general for the diagnosis of nontuberculous mycobacterial infection, mycobacterial culture examination is a time-consuming process. The detection of potentially causative agents directly from clinical samples will aid in practical diagnosis and decision-making for rapid treatment initiation. This is a new laboratory method for species identification, and evaluating its performance is important.

## INTRODUCTION

Mycobacterial diseases are one of the major health concerns worldwide. Although the incidence has been gradually decreasing in recent years, approximately 10 million people newly developed tuberculosis (TB) globally in recent years (1). Additionally, the incidence of nontuberculous mycobacteria (NTM) is increasing in North America (2) and East Asia (3, 4). However, the number of TB disease cases is just the best estimation, with a lower number of bacteriologically confirmed notified cases, and the real prevalence of NTM diseases in other countries is still unknown. For a better understanding of the burden of mycobacterial diseases, rapid and accurate diagnostic methods are essential.

The microbiological diagnosis of mycobacterial diseases is dependent mainly on acid-fast bacillus (AFB) smears and cultures. However, an AFB smear is not species specific and is less sensitive than culture. Moreover, it may take weeks to obtain culture results. The number of species that can be identified is limited, and it requires a sophisticated molecular laboratory if commercial DNA-DNA hybridization (DDH) methods are used for identification, including line probe assays. The use of matrix-assisted laser desorption ionization–time of flight mass spectrometry (MALDI-TOF MS) on mycobacterial isolates enables the more precise identification of a wider variety of species than with DDH; however, MALDI-TOF MS for identification from liquid medium yields poorer results than those using solid medium (5). Thus, clinicians must wait for solid medium culture results to become positive, which takes longer than for cultures in liquid medium, irrespective of the use of MALDI-TOF MS for identification.

Although the nucleic acid amplification test (NAT) is more sensitive than AFB smears and less time-consuming than cultures, the species identified by commercially available NAT kits are limited mainly to Mycobacterium tuberculosis and the Mycobacterium avium/M. intracellulare complex (MAC). The epidemiology of NTM is diverse worldwide. The MAC is generally dominant in North America, East Asia, and other regions, and Mycobacterium abscessus subspecies are secondarily dominant in North America and East Asian regions, including Taiwan (6), whereas Mycobacterium kansasii, Mycobacterium xenopi, and Mycobacterium malmoense are fairly common in some European countries (7). Even within a single country, the distribution of NTM species varies (8, 9). Therefore, mycobacterial NAT kits, which can identify various mycobacterial species comprehensively and simultaneously directly from clinical specimens, can change the laboratory diagnosis of mycobacterial diseases.

The PCR–reverse sequence-specific oligonucleotide probe (PCR-rSSO) method is the DNA typing method commonly used for human leukocyte antigen (HLA) typing (10), in which amplified sample DNA is hybridized to allele-specific arrays and solid-phase probes (11). The PCR-rSSO method-based mycobacterial detection panel (Myco-Panel) test, which was developed by Medical & Biological Laboratories Co., Ltd. (MBL) (Tokyo, Japan), permits the simultaneous identification of 28 mycobacterial species and subspecies, including M. tuberculosis and the MAC, directly from sputum and other lower respiratory samples using the Luminex 100/200 system (Luminex Corp., Austin, TX, USA).

It is expected that various mycobacterial lung diseases can be diagnosed more rapidly and accurately using the Myco-Panel than with the conventional method. Although the PCR-rSSO method is used for antibiotic resistance detection (12–14), along with spoligotyping/subtyping (15–18), and immune response analysis (19) of M. tuberculosis, no previous reports of a PCR-rSSO method-based multiplex detection panel of mycobacteria including M. tuberculosis have been published. Therefore, this study aimed to evaluate the diagnostic performance of the Myco-Panel by performing a fundamental assessment of its detection ability using established mycobacterial strains and conducting a multicenter observational study using clinical samples.

## RESULTS

### Myco-Panel and sequence-based identification results for clinical respiratory samples.

During the study period, 136 patients were included. After the exclusion of 3 patients who could not submit any samples, a total of 178 samples from 133 patients were applied to the Myco-Panel tests: 28 samples from TB cases, 143 samples from NTM cases, 5 samples from suspected mycobacterial infection cases, and 2 samples from bronchitis cases. Of these, 59 samples were sputum samples collected exclusively for this study, 117 were residual samples of sputum collected for culture, and 2 were residual samples of bronchioalveolar lavage (BAL) fluid.

A total of 108 of 178 clinical respiratory samples were positive for mycobacteria by cultures. The species and subspecies were identified by sequencing for 105 positive cultures, but not for 3 due to discrepancies between the sequencing results for several housekeeping genes (Table 1; see also Fig. S2 in the supplemental material).

**TABLE 1:** Clinical respiratory samples^*a*^

Parameter	Value (*n* = 178)
Patients who provided samples	
Median age (yrs) (interquartile range)	69.5 (61–75.8)
No. of patients of sex (%)	
Male	51 (28.7)
Female	127 (71.3)
No. of patients with preexisting diagnosis (%)	
Tuberculosis	28 (15.7)
NTM-LD	143 (80.3)
Suspected mycobacterial disease	5 (2.8)
Bronchitis	2 (1.1)
No. of patients with antimycobacterial medication use (%)	65 (36.5)
No. of samples of type (%)	
Newly collected sputum	59 (33.1)
NALC-processed residual sample of sputum	117 (65.7)
NALC-processed residual sample of BAL fluid	2 (1.1)
No. of samples with AFB smear result (%)	
−	107 (60.1)
−/+	5 (2.8)
1+	53 (29.8)
2+	13 (7.3)
3+	0 (0)
No. of samples with culture- and sequence-based identification result (%)	
Culture negative	70 (39.3)
Mycobacterium tuberculosis	18 (10.1)
Mycobacterium avium	41 (23.0)
Mycobacterium intracellulare	25 (14.0)
Mycobacterium abscessus subsp. *massiliense*	9 (5.1)
Mycobacterium abscessus subsp. *abscessus*	8 (4.5)
Mycobacterium kansasii	1 (0.6)
Mycobacterium gordonae	1 (0.6)
Mycobacterium europaeum	1 (0.6)
Mycobacterium triplex	1 (0.6)
Unidentifiable	3 (1.7)

a NTM-LD, nontuberculous mycobacterial lung disease; NALC, *N*-acetyl-l-cysteine; BAL, bronchoalveolar lavage; AFB, acid-fast bacillus.

The direct Myco-Panel results from clinical respiratory samples and the sequence-based identification results for mycobacterial cultures matched for 148 of 178 samples (concordance, 83.1%; kappa coefficient, 0.785 [95% confidence interval {CI}, 0.716 to 0.854]). The Myco-Panel test detected more than two species in four samples (Table 2). The positive predictive values (PPVs) and negative predictive values (NPVs) for each pathogen detected are shown in Table S1.

**TABLE 2:** Comparison of Myco-Panel results and culture results for clinical respiratory samples

Myco-Panel result(s) for clinical respiratory samples	No. of samples with sequence-based identification result for mycobacterial culture of clinical respiratory samples^*a*^										
	M. tuberculosis	M. avium	M. intracellulare	M. abscessus subsp. *massiliense*	M. abscessus subsp. *abscessus*	M. kansasii	M. gordonae	*M. europaeum*	*M. triplex* ^*b*^	Unidentifiable	Negative
M. tuberculosis	17										
M. avium		38									1
M. intracellulare			21								
M. abscessus subsp. *massiliense*				9							5
M. abscessus subsp. *abscessus*					8						1
M. kansasii						1		1		1	
M. avium and M. intracellulare			1								
M. avium and M. kansasii		1									
M. avium and M. abscessus subsp. *massiliense*		1								1	
Indeterminate			1						1		9
Not detected	1	1	2				1			1	54

a Gray-highlighted cells show that the results of the Myco-Panel and sequence-based identifications in mycobacterial cultures were completely matched.

b *M. triplex* is not covered in the Myco-Panel.

### Myco-Panel and sequence-based identification results for positive liquid cultures.

A total of 108 MGIT-positive samples were also tested by the Myco-Panel. The Myco-Panel and sequence-based identification results matched for 101 samples (concordance rate, 93.5%; kappa coefficient, 0.915 [95% CI, 0.856 to 0.974]) (Table 3 and Table S2).

**TABLE 3:** Comparison of Myco-Panel results and sequence-based identification results for positive culture medium

Myco-Panel result(s) for positive culture medium	No. of samples with sequence-based identification result for positive culture medium^*a*^									
	M. tuberculosis	M. avium	M. intracellulare	M. abscessus subsp. *massiliense*	M. abscessus subsp. *abscessus*	M. kansasii	M. gordonae	*M. europaeum*	*M. triplex* ^*b*^	Unidentifiable
M. tuberculosis	18									
M. avium		40								
M. intracellulare			24							1
M. abscessus subsp. *massiliense*				9						1
M. abscessus subsp. *abscessus*					8					
M. kansasii						1		1		
M. gordonae							1			
M. avium and M. intracellulare			1							
M. avium and M. kansasii		1								1
Indeterminate									1	

a Gray-highlighted cells show that the results of the Myco-Panel and sequence-based identifications of mycobacterial cultures were completely matched.

b *M. triplex* is not covered in the Myco-Panel.

### Myco-Panel and conventional real-time PCR results for clinical respiratory samples.

Real-time PCR assays for M. tuberculosis detection using the Cobas TaqMan MTB (M. tuberculosis) assay were performed for all of the samples, and 17 were positive. This was 100% concordant (kappa coefficient, 1.000) with the Myco-Panel results. The PPV and NPV were 100%.

For the samples with negative Cobas TaqMan MTB assay results, a real-time PCR assay for M. avium and M. intracellulare detection was performed using the Cobas TaqMan MAI (M. avium/M. intracellulare) assay. Of these, 43 samples were positive for M. avium, and 22 samples were positive for M. intracellulare. The rates of concordance with the Myco-Panel were 98.1% (kappa coefficient, 0.952 [95% CI, 0.865 to 0.978]) and 100% (kappa coefficient, 1.000), respectively. The PPV and NPV were 95.3% (95% CI, 84.5% to 98.7%) and 99.2% (95% CI, 95.4% to 99.9%) for M. avium and 100% and 100% for M. intracellulare, respectively (Table 4).

**TABLE 4:** Comparison of the results of the Myco-Panel and a conventional real-time PCR assay for M. tuberculosis and MAC for clinical respiratory samples

Myco-Panel result	No. of samples with Cobas TaqMan assay result		Kappa coefficient, PPV (%), NPV (%) (95% CI)
	Positive	Negative	
M. tuberculosis	Cobas TaqMan MTB (*n* = 178)		
Detected	17	0	1.000, 100, 100
Not detected	0	161	
M. avium	Cobas TaqMan MAI (*n* = 161^*a*^)		
Detected	41	2	0.952 (0.865–0.978), 95.3 (84.5–98.7), 99.2 (95.4–99.9)
Not detected	1	117	
M. intracellulare	Cobas TaqMan MAI (*n* = 161^*a*^)		
Detected	22	0	1.000, 100, 100
Not detected	0	139	

a The Cobas TaqMan MAI assay was not performed for the samples that were positive by the Cobas TaqMan MTB assay.

### Myco-Panel results for stock mycobacterial strains.

Of the 283 strains, including 70 type strains and 213 clinical isolates, that were used for the analysis, 231 strains/isolates were identified by the Myco-Panel, but the other 50 strains were not (Fig. S3). The species of these 50 strains/isolates were out of the Myco-Panel targets. As a result, the Myco-Panel correctly identified 281 strains, while only 1 M. intracellulare strain and 1 M. kansasii strain were not correctly detected. The concordance rate was 99.3% (kappa coefficient, 0.992 [95% CI, 0.982 to 1.000]) (Table 5). The PPVs and NPVs for each pathogen detected were no less than 99% for all species, except for the PPV of M. avium (kappa coefficient, 91.7% [95% CI, 74.2% to 97.7%]) (Table S3).

**TABLE 5:** Myco-Panel results for stock mycobacterial strains

Myco-Panel result(s) for clinical respiratory samples	No. of samples with result for frozen stocks of mycobacterial type strains and clinically isolated strains^*c*^																													
	M. tuberculosis	M. tuberculosis subsp. BCG	M. avium	M. intracellulare	M. kansasii	M. abscessus subsp. *abscessus*	M. abscessus subsp. *bolletii*	M. abscessus subsp. *massiliense*	M. chelonae	M. gordonae	M. xenopi	M. fortuitum	*M. szulgai*	M. marinum	M. ulcerans	M. scrofulaceum	M. simiae	*M. asiaticum*	*M. lentiflavum*	*M. nonchromogenicum*	*M. shimoidei*	*M. terrae*	*M. shinjukuense*	M. mucogenicum	*M. peregrinum*	*M. triviale*	*M. malmoense*	*M. chimaera*	*M. heckeshornense*	Species not covered in Myco-Panel^*d*^
M. tuberculosis	24																													
M. tuberculosis subsp. BCG		1																												
M. avium			22		1																									
M. intracellulare				21																										
M. avium + M. intracellulare				1																										
M. kansasii					12																									
M. abscessus subsp. *abscessus*						21																								
M. abscessus subsp. *bolletii*							2																							
M. abscessus subsp. *abscessus*/*bolletii*^*a*^						1																								
M. abscessus subsp. *massiliense*								21																						
M. chelonae									6																					
M. gordonae										21																				
M. xenopi											4																			
M. fortuitum												23																		
*M. szulgai*													6																	
M. marinum/M. ulcerans^*b*^														5	1															
M. scrofulaceum																1														
M. simiae																	5													
*M. asiaticum*																		1												
*M. lentiflavum*																			6											
*M. nonchromogenicum*																				1										
*M. shimoidei*																					4									
*M. terrae*																						1								
*M. shinjukuense*																							5							
M. mucogenicum																								3						
*M. peregrinum*																									4					
*M. triviale*																										3				
*M. malmoense*																											2			
*M. chimaera*																												1		
*M. heckeshornense*																													3	
Indeterminate																														41
Not detected																														9

a M. abscessus strains that could be distinguished from M. abscessus subsp. *massiliense* but could not be determined as M. abscessus subsp. *bolletii* or M. abscessus subsp. *abscessus* were identified as M. abscessus subsp. *abscessus*/*bolletii* in the Myco-Panel analysis.

b The Myco-Panel could not distinguish M. marinum from M. ulcerans.

c Clinically isolated strains were originally identified by the DNA-DNA hybridization (DDH) method. In cases where the DDH-based identification results and the Myco-Panel results were discrepant, reidentification by sequencing of the housekeeping genes was performed. Gray-highlighted cells show that the results were completely matched.

d Species not covered in the Myco-Panel included 36 type stains of *M. intermedium*, *M. triplex*, *M. neworleansense*, *M. bohemicum*, *M. conspicuum*, M. porcinum, *M. nebraskense*, *M. lacus*, M. smegmatis, *M. parascrofulaceum*, *M. heidelbergense*, *M. brisbanense*, *M. interjectum*, *M. sherrisii*, *M. conceptionense*, *M. palustre*, *M. goodie*, *M. houstonense*, *M. parmense*, *M. mageritense*, *M. neoaurum*, *M. saskatchewanense*, *M. thermoresistibile*, *M. novocastrense*, *M. branderi*, *M. boenickei*, *M. senegalense*, *M. celatum*, M. canariasense, *M. wolinskyi*, M. genavense, *M. elephantis*, *M. gastri*, M. haemophilum, *M. immunogenum*, and M. marseillense; clinically isolated strains of *M. celatum*, *M. kyorinense*, *M. nebraskense*, *M. parascrofulaceum*, *M. europaeum*, *M. kumamotonense*, *M. aubagnense*, *M. moriokaense*, and *M. koreensis* (identified by sequencing of the housekeeping genes); and mycobacterial strains unidentifiable by sequencing.

## DISCUSSION

For the proper management of patients and infection control, the rapid detection and accurate identification of NTM are important in countries where lung diseases due to many species of NTM are more prevalent than TB. While the identification of mycobacteria has recently been performed by MALDI-TOF MS, it is still necessary to obtain mycobacterial colonies recovered on solid media. In the current study, the diagnostic capacity of the Myco-Panel, using stocked mycobacterial strains and positive MGIT media from clinical samples, demonstrated satisfactory accuracy compared to that of sequence-based identification, demonstrating that the Myco-Panel could work as a rapid diagnostic/identification tool for the species commonly isolated around the world.

The direct application of the Myco-Panel to respiratory samples demonstrated >80% concordance with culture- and sequence-based identifications. As for the seven samples that were positive according to the Myco-Panel, their cultures were negative, even when five of them were positive by an AFB smear. They were obtained from confirmed NTM lung disease cases, and some of the patients were on antimycobacterial therapy, which could explain the false-negative results from culture, resulting in the discrepancy. In fact, we found the records of the positive culture results for the species detected by the Myco-Panel according to a chart reviews of these discrepant cases on antimycobacterial therapy.

NTM coinfection with TB is often reported. De Keukeleire et al. (20) reported that 35 NTM coinfection cases were found among 2,569 culture-positive pulmonary TB cases in a multicenter, retrospective cohort study in Belgium. Khan et al. (21) reported 5 patients coinfected with MAC isolates and TB, who were included among 89 patients with at least one respiratory culture positive for MAC isolates at two hospitals in the United States. In Japan, nationwide surveillance for drug-resistant TB in 2002 revealed that 19.3% of the mycobacterial strains recovered from sputum samples collected from culture-confirmed TB patients were mycobacteria other than M. tuberculosis, which indicates that mixed infections of TB and NTM are common (22). In order to detect such coinfection cases and perform careful laboratory procedures to detect multiple species from mycobacterial cultures, NATs detecting TB and NTM simultaneously are important.

Furthermore, NATs detecting TB and NTM simultaneously are also important for countries where TB is highly prevalent and where empirical TB treatment is initiated according to a presumptive diagnosis of TB. Aliyu et al. (23) performed mycobacterial cultures of sputum samples collected from 1,603 patients who were given a presumptive diagnosis of TB according to symptoms in Nigeria, which is one of five countries in the world where TB is highly prevalent. Among the 444 patients with positive mycobacterial cultures, NTM were recovered from 15% of them. Bonnet et al. (24) reported that 124 of 1,183 patients with presumptive TB in Cambodia had NTM isolated from sputum cultures. These findings imply that in developing countries with a high TB prevalence, although it could be contamination/colonization, substantial numbers of NTM patients are misdiagnosed with TB and empirically prescribed TB treatments. NATs detecting TB and NTM simultaneously are also important for developing countries with a high TB prevalence to improve clinical practice and the quality of the epidemiology for the management of patients with mycobacteriosis.

Although NATs detecting TB and NTM simultaneously have already been developed, the number available is limited. Sarro et al. (25) developed multiplex PCR test kits to allow the simultaneous detection of M. tuberculosis, MAC, and other NTM and reported that its accuracy was comparable to that of the Xpert MTB/RIF assay, which is endorsed by the World Health Organization as a NAT for TB diagnosis (26). However, the kits could not identify NTM species other than the MAC; thus, more time-consuming processes were required to diagnose NTM disease caused by other species.

Chen et al. (27) developed a system for the simultaneous detection of the Mycobacterium genus and identification of TB using multiplex PCR in combination with a denaturing high-performance liquid chromatography system named Transgenomic Wave 450 (Precipio, Omaha, NE, USA), which demonstrated the highly accurate detection of 23 mycobacterial species from clinical samples. However, it was not confirmed whether the system can simultaneously identify subspecies of Mycobacterium abscessus, which is critical for clinicians because inducible resistance to macrolides conferred by *erm*(41) leads to poor outcomes in M. abscessus subsp. *abscessus* and M. abscessus subsp. *bolletii* infections (28). In this regard, the Myco-Panel multiplex assay successfully distinguished M. abscessus subsp. *abscessus* and M. abscessus subsp. *bolletii* from M. abscessus subsp. *massiliense* in the analysis using both clinical respiratory samples and stock strains.

In this study, the Myco-Panel detected two mycobacterial species from the respiratory samples of four patients. A retrospective chart review revealed that all of these patients had positive culture results for two species simultaneously or at different times previously, all of which were detected by the Myco-Panel. Thus, it might not be false-positive detections of mixed infections by the Myco-Panel in patients with a single pathogen, but it might be the more sensitive detection of active or inactive mixed infection cases than with culture-based methods.

This study had three limitations. First, only MGIT-based cultures were performed for the culture method. Therefore, if mixed pathogens were included in the samples, the nucleic acid extracts might include genes derived from two or more species, which might lead to unidentifiable results in housekeeping gene sequence-based identifications. Therefore, confirmation of clonality using a solid medium might be better. Second, frozen stocks of clinical isolates were identified by DDH methods only if the Myco-Panel results agreed with the DDH results. Therefore, the accuracy of this form of identification used as a control is limited (29). Finally, according to the epidemiology of mycobacteria in Japan, the number of samples or strains with species rarely identified in Japan is limited. Therefore, data regarding PPVs and the possibility of cross-hybridization are insufficient. Furthermore, in our study, samples contaminated with blood or other materials that might interfere with the PCRs did not exist. Therefore, PCR inhibition and potential assay interference were unclear.

In conclusion, the novel PCR-rSSO method-based mycobacterial detection panel test could accurately detect a variety of mycobacterial species, including TB, from clinical respiratory samples and mycobacterial suspensions.

## MATERIALS AND METHODS

### Clinical respiratory samples.

From 3 September 2018 to 31 January 2020, samples for this multicenter, observational study were collected at three hospitals in the Tokyo area: Fukujuji Hospital, Japan Anti-Tuberculosis Association (JATA); National Hospital Organization (NHO) Tokyo National Hospital; and Keio University Hospital.

Outpatients or inpatients from these three hospitals who fulfilled all of the following criteria were recruited to the study: (i) patients had confirmed or suspected pulmonary mycobacterial infections or other respiratory illnesses; (ii) sputum, residual samples of pretreated sputum, or bronchoalveolar lavage (BAL) fluid could be obtained; (iii) written informed consent could be obtained from the patients or their proxies; and (iv) patients were aged 20 years or older. Patients who fulfilled any of the following criteria were excluded: (i) patients whose mycobacterial cultures were suspected to have become negative under antimycobacterial chemotherapy, (ii) patients whose samples were inappropriate for the study (i.e., salivary samples without secretions from the lower respiratory tract), and (iii) TB patients who received anti-TB chemotherapy for more than 2 weeks.

One or two newly collected sputum samples or *N*-acetyl-l-cysteine sodium hydroxide-processed residual samples of sputum or BAL fluid were obtained from each participant and used for the study.

The study protocol was approved by the ethics committee of each hospital (IRB approval numbers 19031 [Fukujuji Hospital, JATA], 30-08-Hea [NHO Tokyo National Hospital], and 20180080 [Keio University Hospital]), and written informed consent was obtained from all of the participants.

### PCR-rSSO method-based mycobacterial detection panel test (Myco-Panel).

Nucleic acids were extracted from clinical respiratory samples using Amplicor mycobacterial sample processing kit II (Roche Diagnostics KK, Tokyo, Japan). Extracts (10 μL) were tested with a Myco-Panel named the Mebgen mycobacterial nucleic acid identification kit. Mycobacterial 16S rRNA, *sod*, RD1, the 16S-23S internal transcribed spacer (ITS) region, and indel A and indel B regions were amplified by multiplex PCR using *Taq* DNA polymerase and biotin-labeled specific primers. After the addition of a species-specific-probe-tagged bead mix to amplicons for hybridization, a streptavidin-phycoerythrin conjugate was added, the fluorescence intensity was measured by using the Luminex 100/200 system, and the species were identified by the pattern of fluorescence. Detailed methods for panel analysis are shown in Fig. S1 in the supplemental material.

The Myco-Panel can identify Mycobacterium tuberculosis var. *tuberculosis*, Mycobacterium tuberculosis var. BCG, Mycobacterium avium, Mycobacterium intracellulare, Mycobacterium kansasii, Mycobacterium abscessus subsp. *abscessus*, Mycobacterium abscessus subsp. *bolletii*, Mycobacterium abscessus subsp. *massiliense*, Mycobacterium chelonae, Mycobacterium gordonae, Mycobacterium xenopi, Mycobacterium fortuitum, Mycobacterium szulgai, Mycobacterium marinum/Mycobacterium ulcerans, Mycobacterium scrofulaceum, Mycobacterium simiae, Mycobacterium asiaticum, Mycobacterium lentiflavum, Mycobacterium nonchromogenicum, Mycobacterium shimoidei, Mycobacterium terrae, Mycobacterium shinjukuense, Mycobacterium mucogenicum, Mycobacterium peregrinum, Mycobacterium triviale, Mycobacterium malmoense, Mycobacterium chimaera, and Mycobacterium heckeshornense.

### Culture of samples and identification of strains.

Clinical respiratory samples were cultured in broth culture medium using an MGIT system (Becton, Dickinson, Franklin Lakes, NJ, USA). In cases where cultures turned positive within 6 weeks, nucleic acids were extracted from positive medium using the DNeasy blood and tissue kit (Qiagen, Hilden, Germany). Sequencing of the 16S rRNA, *hsp65*, *rpoB*, and *dnaJ* genes was performed using primer sequences and conditions described previously (30–33). Sequencing data were analyzed using the BLAST database, and the mycobacterial species were identified by homologies of ≥98.7%. When M. abscessus was identified, subspecies were identified by a PCR assay using primers specific for each subspecies as described in a previous report and agarose gel electrophoresis of the amplicon (34). The multisequence-based mycobacterial identification results were compared with the results of the Myco-Panel tests. Furthermore, to assess the identification ability of the Myco-Panel, Myco-Panel tests were also performed for nucleic acid extraction from the positive MGIT tubes.

### Comparison with a conventional real-time PCR assay.

To determine the diagnostic accuracy of the Myco-Panel regarding TB and MAC detection, a conventional commercial real-time PCR assay was performed on the clinical respiratory samples. The Cobas TaqMan MTB and Cobas TaqMan MAI assays (Roche Diagnostics KK, Tokyo, Japan) were used with the Cobas TaqMan 48 real-time PCR system (Roche Diagnostics KK, Tokyo, Japan), according to the manufacturer’s instructions, which can detect M. tuberculosis, M. avium, and M. intracellulare. We compared the Myco-Panel results with the Cobas TaqMan MTB and MAI assay results.

### Frozen stocks of strains of mycobacteria.

The variety of mycobacterial species recovered from clinical respiratory samples was limited to the mycobacterial epidemiology in Japan. Thus, to assess the detection performance of the Myco-Panel test for a variety of mycobacterial species, including those rarely recovered in Japan, Myco-Panel tests were performed on frozen stocks of mycobacterial strains, including type strains and clinical isolates. Seventy type strains of 65 mycobacterial species and subspecies and 223 clinical isolates of 24 mycobacterial species and subspecies were included; these were cultured in solid culture medium, and DNA was extracted using the Isoplant kit (Nippon Gene Co., Ltd.). Extracts were adjusted to a concentration of 1 × 10^5^ copies/μL, and 10 μL of the concentration-adjusted extracts was used for the Myco-Panel test. Clinical isolates were identified mainly using DDH mycobacterial Kyokuto test kits (Kyokuto Pharmaceutical Industrial Co., Ltd., Tokyo, Japan) according to the manufacturer’s instructions (35). When the results of DDH were not concordant with those of the Myco-Panel tests, the isolates were reidentified by the sequencing method described above. The clinical isolates that could not be identified by sequencing were excluded from the analysis. Furthermore, the identification of subspecies was performed for Mycobacterium abscessus.

### Statistical analyses.

For comparison of the Myco-Panel results to the results of the reference method, overall concordance and Cohen’s kappa coefficient values were calculated. Myco-Panel results that were completely identical to those of the reference method were considered concordant. Even if one of the multiple pathogens detected by the Myco-Panel was detected by the reference method, such partially matched results were treated as discrepant. Kappa coefficients were interpreted according to the criteria proposed previously by Byrt; i.e., a value of 1.00 to 0.93 is excellent, 0.92 to 0.81 is very good, 0.80 to 0.61 is good, 0.60 to 0.41 is fair, 0.40 to 0.21 is slight, 0.20 to 0.01 is poor, and <0.00 indicates no agreement (36). Furthermore, positive predictive values (PPVs), negative predictive values (NPVs), and Cohen’s kappa coefficients, including their 95% confidence intervals, for the detection of each pathogen were also assessed. For statistical analysis, SAS 9.4 software and JMP 16.0 software (SAS Institute, Cary, NC, USA) were used.
