# Supplementary material for: Diagnostic Utility of a Mycobacterium Multiplex PCR Detection Panel for Tuberculosis and Nontuberculous Mycobacterial Infections
Source: Microbiol Spectr. 2023 Apr 24;11(3):e05162-22. doi: 10.1128/spectrum.05162-22 (PMC10269564; doi:10.1128/spectrum.05162-22)
Supplement: Supplemental file 1 — Supplemental material. Download spectrum.05162-22-s0001.pdf, PDF file, 0.5 MB [file spectrum.05162-22-s0001.pdf]

Supplemental materials

Figure S1. Detailed methods of Myco-Panel analysis

DNA extraction

- DNA extraction were performed using the following commercial kits according to the manufacturer's instructions.
  - Clinical respiratory samples; the Amplicore mycobacteria sample processing kit II (Roche Diagnostics K.K., Tokyo, Japan).
  - MGIT positive samples; DNeasy® Blood & Tissue（QIAGEN, Hilden, Germany）
  - Frozen-stocked strains; ISOPLANT (NIPPON GENE CO. LTD., Tokyo, Japan)

Amplification

- 10 µL volume of DNA extractions were mixed with Taq DNA polymerase and biotin-labeled-specific primers (number of primers and regions are displayed in a right table; primer sequences are confidential).
- The mixture were amplified using Gold 96-well GeneAmp PCR System 9700 or Applied Biosystems VRTi Dx (ThermoFisher Scientific, Waltham, MA, USA) according to the following conditions;

  Hold 40 °C 5 minutes
  Hold 95 °C 2 minutes
  95 °C 20 seconds – 63 °C 30 seconds – 72 °C 30 seconds (60 Cycles)
  Hold 72 °C 1 minutes
  Hold 95 °C 10 minutes

Region	Number of primers	
	Forward	Reverse
16S rRNA	17	1
sod	5	7
16S-23 ITS	1	1
Indel A/B	2	6
RD1	1	1
SIC	1	1

SIC: sample independent control for quality control of the assay.

Hybridization

- 5 µL volume of species-specific probe tagged beads mix and 40 µL volume of hybridization buffer were mixed with 5 µL volume of amplicons.
- The mixture were hybridized using Gold 96-well GeneAmp PCR System 9700 or Applied Biosystems VRTi Dx (ThermoFisher Scientific, Waltham, MA, USA) according to the following conditions;

  Hold 95 °C 2 minutes
  Hold 52 °C 30 minutes

Fluorescence measurement

- After hybridization, beads were washed and streptavidin-phycoerythrin conjugate was added.
- Fluorescent intensity were measured by Luminex 100/200 System (Luminex Corp, Austin, TX, USA). Fluorescent cutoffs were different in each beads. According to the positive/negative patterns of the beads, mycobacterial species were identified.

Figure S2. Diagrams about the samples used in the analysis

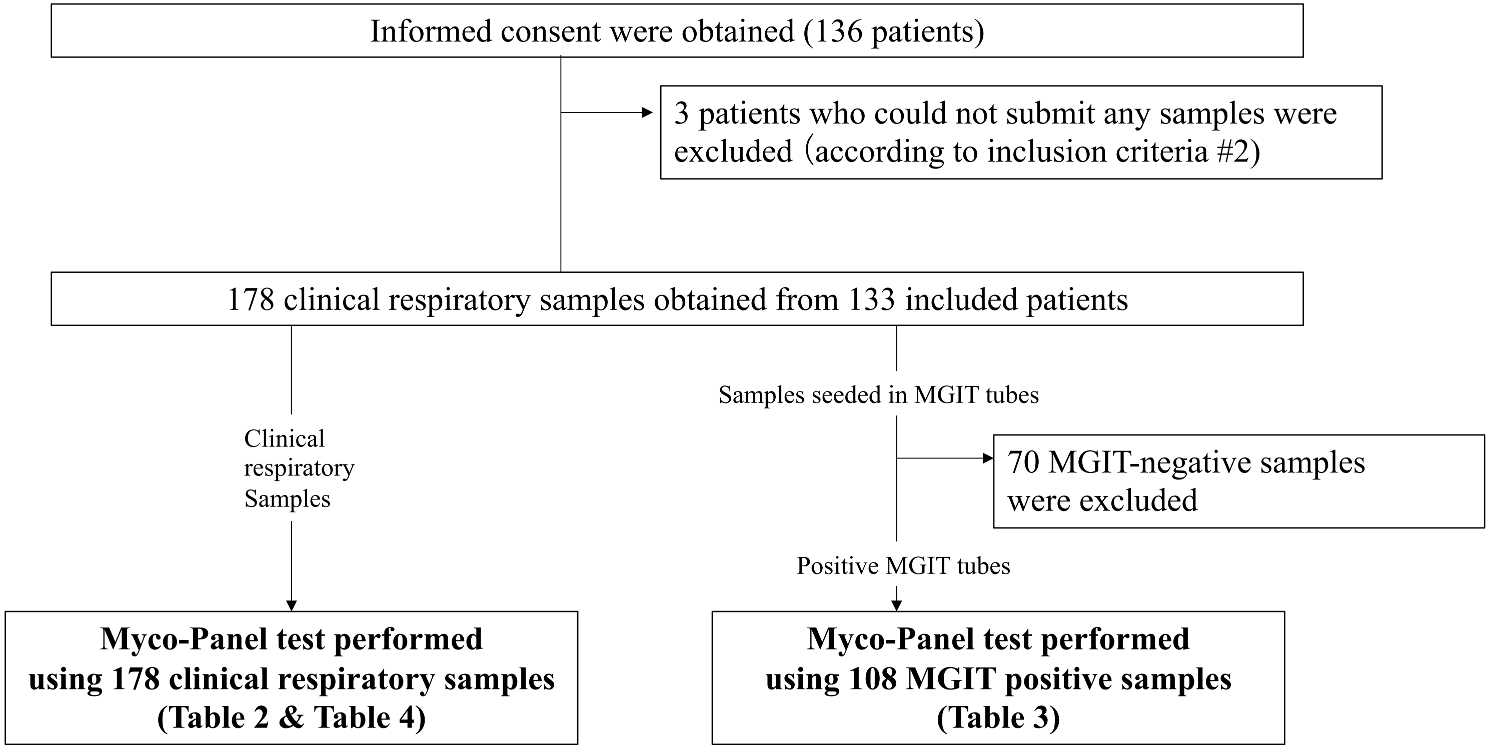

Figure S3. Diagrams about the stocked strains used in the analysis

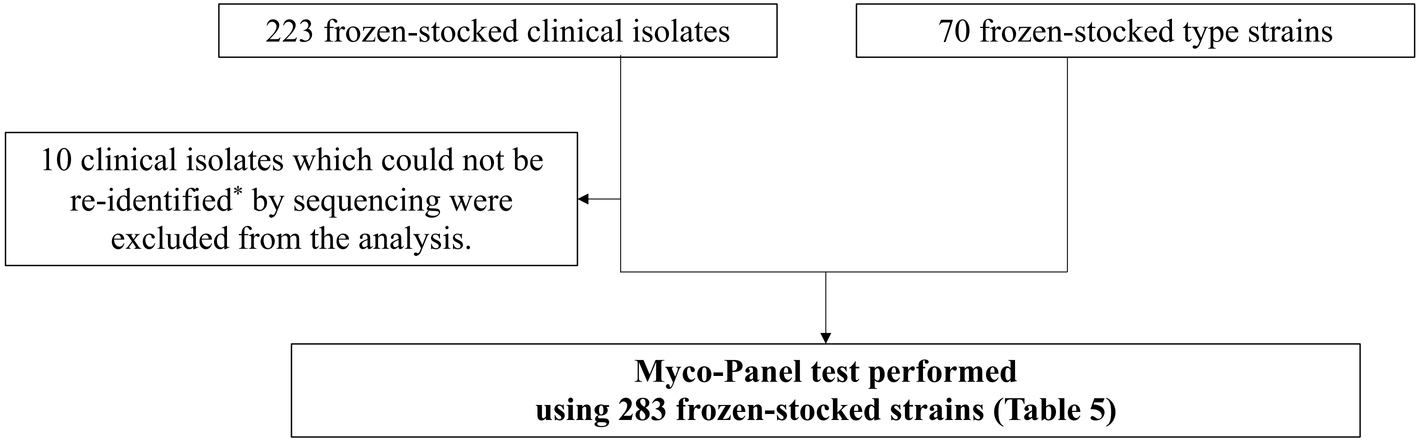

*When the identification of DDH were not concordant with those of the Myco-Panel tests, the isolates were re-identified by the sequencing method

Table S1. Comparison of Myco-Panel results and culture results for clinical respiratory samples by each pathogen detection

Species	Myco-Panel results	Sequence-based identification results of culture		PPV (%) NPV (%)		(95% CI)	Kappa	(95% CI)
		Detected	Not-detected					
M. tuberculosis	Detected	17	0	PPV	100	NA	0.968	(0.906–1.000)
	Not-detected	1	160	NPV	99.4	(96.6–99.9)		
M. avium	Detected	40	2	PPV	95.2	(84.2–98.7)	0.953	(0.900–1.000)
	Not-detected	1	135	NPV	99.3	(96.0–99.9)		
M. intracellulare	Detected	22	0	PPV	100	NA	0.926	(0.844–1.000)
	Not-detected	3	153	NPV	98.1	(94.5–99.3)		
M. abscessus* subsp. *massiliense	Detected	9	6	PPV	60.0	(35.2–84.8)	0.733	(0.531–0.935)
	Not-detected	0	163	NPV	100	NA		
M. abscessus* subsp. *abscesssus	Detected	8	1	PPV	88.9	(56.5–98.0)	0.938	(0.818–1.000)
	Not-detected	0	169	NPV	100	NA		
M. kansasii	Detected	1	3	PPV	25.0	(4.6–69.9)	0.395	(-0.146–0.935)
	Not-detected	0	174	NPV	100	NA		
M. gordonae	Detected	0	0	PPV	–	–	—	—
	Not-detected	1	177	NPV	—	—		
***M. europaeum*†**	Detected	0	0	PPV	–	–	—	—
	Not-detected	1	177	NPV	—	—		
***M. triplex*†**	Detected	0	0	PPV	–	–	—	—
	Not-detected	1	177	NPV	—	—		

†*M. europaeum* and *M. triplex* are not covered in the Myco-Panel

Table S2. Comparison of Myco-Panel results and sequence-based identification results of positive culture medium by each pathogen detection

Species	Myco-Panel results	Sequence-based identification results of culture		PPV (%) NPV (%)		(95% CI)	Kappa	(95% CI)
		Detected	Not-detected					
M. tuberculosis	Detected	18	0	PPV	100	NA	1.000	NA
	Not-detected	0	90	NPV	100	NA		
M. avium	Detected	41	1	PPV	97.6	(87.7–99.6)	0.980	(0.942–1.000)
	Not-detected	0	66	NPV	100	NA		
M. intracellulare	Detected	25	0	PPV	100	NA	0.974	(0.924–1.000)
	Not-detected	1	82	NPV	98.8	(93.5–99.8)		
M. abscessus* subsp. *massiliense	Detected	9	1	PPV	90.0	(59.6–98.2)	0.942	(0.830–1.000)
	Not-detected	0	98	NPV	100	NA		
M. abscessus* subsp. *abscesssus	Detected	8	0	PPV	100	NA	1.000	NA
	Not-detected	0	100	NPV	100	NA		
M. kansasii	Detected	1	2	PPV	33.3	(6.1–79.2)	0.493	(-0.107–1.000)
	Not-detected	0	105	NPV	100	NA		
M. gordonae	Detected	1	0	PPV	100	NA	1.000	NA
	Not-detected	0	107	NPV	100	NA		
***M. europaeum*†**	Detected	0	0	PPV	–	–	–	–
	Not-detected	1	107	NPV	–	–		
***M. triplex*†**	Detected	0	0	PPV	–	–	–	–
	Not-detected	1	107	NPV	–	–		

†*M. europaeum* and *M. triplex* are not covered in the Myco-Panel

Table S3. Myco-Panel results for Stocked Mycobacteria Strains by each pathogen detection

Species	Stocked Mycobacteria Strains**									
	Included		Not-Included							
Myco-Panel results	Detected	Not -detected	Detected	Not -detected	PPV (%)	(95% CI)	NPV (%)	(95% CI)	Kappa	(95% CI)
M. tuberculosis	24	0	0	259	100	NA	100	NA	1.000	NA
***M. tuberculosis* var. BCG**	1	0	0	282	100	NA	100	NA	1.000	NA
M. avium	22	0	2	259	91.7	(74.2–97.7)	100	NA	0.953	(0.887–1.000)
M. intracellulare	22	0	0	261	100	NA	100	NA	1.000	NA
M. kansasii	12	1	0	270	100	NA	99.6	(97.9–99.9)	0.958	(0.876–1.000)
M. abscessus* subsp. *abscessus/bolletii*	24	0	0	259	100	NA	100	NA	1.000	NA
M. abscessus* subsp. *massiliense	21	0	0	262	100	NA	100	NA	1.000	NA
M. chelonae	6	0	0	277	100	NA	100	NA	1.000	NA
M. gordonae	21	0	0	262	100	NA	100	NA	1.000	NA
M. xenopi	4	0	0	279	100	NA	100	NA	1.000	NA
M. fortuitum	23	0	0	260	100	NA	100	NA	1.000	NA
M. szulgai	6	0	0	277	100	NA	100	NA	1.000	NA
***M. marinum/ulcerans*†**	6	0	0	277	100	NA	100	NA	1.000	NA
M. scrofulaceum	1	0	0	282	100	NA	100	NA	1.000	NA
M. simiae	5	0	0	278	100	NA	100	NA	1.000	NA
M. asiaticum	1	0	0	282	100	NA	100	NA	1.000	NA
M. lentiflavum	6	0	0	277	100	NA	100	NA	1.000	NA
M. nonchromogenicus	1	0	0	282	100	NA	100	NA	1.000	NA
M. shimoidei	4	0	0	279	100	NA	100	NA	1.000	NA
M. terrae	1	0	0	282	100	NA	100	NA	1.000	NA
M. shinjukuense	5	0	0	278	100	NA	100	NA	1.000	NA
M. mucogenicum	3	0	0	280	100	NA	100	NA	1.000	NA
M. peregrinum	4	0	0	279	100	NA	100	NA	1.000	NA
M. trivialis	3	0	0	280	100	NA	100	NA	1.000	NA
M. malmoense	2	0	0	281	100	NA	100	NA	1.000	NA
M. chimaera	1	0	0	282	100	NA	100	NA	1.000	NA
M. heckeshornense	3	0	0	280	100	NA	100	NA	1.000	NA

*In this table, *M. abscessus subsp abscessus/bolletii* strains included *M. abscessus* subsp. *bolletii* strains, *M. abscessus* subsp. *abscessus* strains, and *M. abscessus* strains which could be distinguished from *M. abscessus* subsp. *massiliense* but could not be determined *M. abscessus* subsp. *bolletii* or *M. abscessus* subsp. *abscessus* in the Myco-Panel analysis.

†The Myco-Panel could not distinguish *M. marinum* from *M. ulcerans*.

**Stocked Mycobacteria strains included type strains and clinically isolated strains. Clinically isolated strains were originally identified by DNA-DNA hybridization (DDH) method. In cases where the DDH-based identification results and Myco-Panel results were discrepant, re-identification by sequencing of house-keeping genes was performed.
